# Financing entrepreneurship in times of crisis: Exploring the impact of COVID-19 on the market for entrepreneurial finance in the United Kingdom

**DOI:** 10.1177/0266242620937464

**Published:** 2020-08

**Authors:** Ross Brown, Augusto Rocha, Marc Cowling

**Affiliations:** University of St Andrews, UK; University of St Andrews, UK; University of Derby, UK

**Keywords:** COVID-19, crisis, entrepreneurial finance, entrepreneurship, seed finance

## Abstract

This commentary explores the manner in which the current COVID-19 crisis is affecting key sources of entrepreneurial finance in the United Kingdom. We posit that the unique relational nature of entrepreneurial finance may make it highly susceptible to such a shock owing to the need for face-to-face interaction between investors and entrepreneurs. The article explores this conjecture by scrutinising a real-time data source of equity investments. Our findings suggest that the volume of new equity transactions in the United Kingdom has declined markedly since the outbreak of the COVID-19 pandemic. It appears that seed finance is the main type of entrepreneurial finance most acutely affected by the crisis, which typically goes to the most nascent entrepreneurial start-ups facing the greatest obstacles obtaining finance. Policy makers can utilise these real-time data sources to help inform their strategic policy interventions to assist the firms most affected by crisis events.

## Introduction

This commentary explores the impact the COVID-19 pandemic is having upon entrepreneurial activity in the United Kingdom by examining how the unfolding crisis is affecting the market for entrepreneurial finance. Policy maker attention has inevitably, and quite understandably, centred on the immediate effects the COVID-19 crisis has for existing small and medium-sized enterprises (SMEs) in terms of their ability to maintain staffing levels, avoid cash-flow problems and prevent widespread bankruptcies in the wake of the lockdown (Organisation for Economic Co-operation and Development (OECD), 2020). Empirical work from around the world shows that as many as half of all small firms have temporarily ceased trading since the lockdown and as many as 60% of SMEs are at risk of running out of their cash reserves (Bartik et al., in press; Cowling et al., 2020; Giupponi and Landais, 2020). While mitigating the immediate aftershocks of the COVID-19 crisis is crucial for the short-term stability of the economy, we wish to look at a longer-term indicator of entrepreneurial activity – entrepreneurial finance – and how this has, and will be, affected.

Finance is crucial for start-ups (Cassar, 2004). However, owing to the informationally opaque nature of innovative growth-oriented start-ups, such firms are often deemed too risky and unsuitable for bank finance due to their lack of collateral and unstable cash-flows (Berger and Udell, 1998). Typically, these types of firms seek recourse to entrepreneurial sources of finance from business angels (BAs) and venture capitalists (VCs) (Hall and Lerner, 2010; Kerr et al., 2014). This type of finance is particularly salient for high-growth firms as they are more likely to use equity finance than non-high-growth firms (British Business Bank, 2020; Brown and Lee, 2019).

Outside equity investors not only contribute financial capital to aid rapid firm growth, they also bring ancillary benefits and added value through their experience, expertise and access to networks for the recipients of these investments (Bernstein et al., 2016). Entrepreneurial finance is also viewed as a vital means of facilitating blockbuster entrepreneurship in the form of scale-ups (Cumming et al., 2018). Therefore, how the uncertainty caused by the crisis affects the market for entrepreneurial finance will have a strong bearing on the levels of entrepreneurial dynamism and innovation within the UK economy for years to come.

Our starting point is that the literature has struggled to fully comprehend how entrepreneurial activity is upended, mediated and re-aligned by crisis episodes (Doern et al., 2019; Herbane, 2010; Wenzel et al., 2020). Research on the impact of crisis events for SMEs is sparse, despite the fact that SMEs are often the firms most disadvantaged by crisis episodes (Doshi et al., 2018). There is also a dearth of research on entrepreneurial resilience and crisis management as a whole within the context of SMEs (Herbane, 2013; Wishart, 2018). Yet, initial work suggests that the gravity of the COVID-19 crisis is such (Baker et al., 2020) that it could potentially be wreaking such devastating economic and societal consequences we may be witnessing the greatest crisis period facing humankind since the World War II.^1^ Such is the uniqueness of the current crisis; some label it a metaphorical ‘Black Swan event’ for entrepreneurship (Kuckertz et al., 2020), as it encompasses virtually every sector and every country spanning the entire global economy simultaneously (Goodell, 2020).

While research emphatically suggests access to bank finance becomes more problematic for innovative firms during previous crisis episodes such as the global financial crisis (GFC) (Cowling et al., 2012; Demirgüç-Kunt et al., 2020; Lee et al., 2015), much less evidence exists for how these shock events influence the market for entrepreneurial sources of finance from VCs and BAs (Block and Sandner, 2009; Conti et al., 2019). VC is very volatile which makes this form of investment highly susceptible to the uncertainty caused by shock events (Gompers et al., 2008) and some have speculated that entrepreneurial finance may be especially affected by the current pandemic (Brown and Rocha, 2020).

Unlike debt finance, equity funding is strongly predicated on the need for close personal engagement between investors and entrepreneurs (De Clercq and Sapienza, 2006). A key aspect of the relational interaction is the oral ‘pitch’ entrepreneurs undertake to secure an investment from investors (Clark, 2008). As Huang and Knight (2017) note, other important relational interactions or ‘dates’ between investors and entrepreneurs such as impromptu social meetings for coffee also emphasise crucial parts of the investment decision-making process. Investors know that every entrepreneur has strengths and weaknesses so personal knowledge and closely ‘vetting’ the individuals concerned to generate soft information reduces the informational opacity associated with start-ups (Shane and Cable, 2002). These intimate relationships are vital for equity investors because they rely heavily upon ‘personal networks and face-to-face contacts in finding, evaluating, and monitoring investment opportunities’ (Martin et al., 2005: 1213).

Given that relationships patently matter within the market for sources of entrepreneurial finance, shocks such as the current COVID-19 pandemic could fundamentally disrupt this form of finance (Howell et al., 2020). That said, the upsurge in technological development means that much more risk finance is now allocated by investors via online equity crowdfunding platforms (Brown et al., 2018; Fraser et al., 2015; Nesta, 2019). Indeed, a recent major survey revealed that almost half (45%) of all UK angels invested via equity crowdfunding platforms (Wright et al., 2015). Given that investors are also increasingly familiar with online ‘video pitches’ used by entrepreneurs to obtain equity finance via crowdfunding platforms, perhaps these trends will mitigate the need to physically meet to engage with investors during events such as the COVID-19 crisis.

The setting for this study is the United Kingdom which has the largest (40% of the European total)^2^ market and associated ecosystem for entrepreneurial finance in Europe, both in terms of volumes and value of deals (Bertoni et al., 2015; British Business Bank, 2020). Furthermore, the rapid growth of equity finance has dramatically increased the number of providers of entrepreneurial finance (such as incubators, accelerators, BAs and equity crowdfunding) within the United Kingdom since the GFC (Bonini and Capizzi, 2019) which in turn may have increased resilience levels within the United Kingdom’s equity funding ecosystem to the current crisis. Economists argue that to effectively estimate the current and future effects of COVID-19 induced uncertainties, we need measures of uncertainty that are available in real time (Baker et al., 2020). To explore how the COVID-19 crisis is affecting sources of entrepreneurial finance in the United Kingdom, in line with others (Block and Sandner, 2009), we examine a novel real-time data source provided by Crunchbase. These instantaneous real-time data sources are becoming increasingly prevalent within entrepreneurship research (Schwab and Zhang, 2019).

Crunchbase data are derived from 12,259 funding transactions that raised over $40 billion in the United Kingdom between January 2007 and April 2020. It uses a range of data providers and techniques, including a global network of investment firms, community contributors (e.g. investors, entrepreneurs), data analysts, artificial intelligence and machine learning algorithms,^3^ to distribute company data practically in real time, including funding rounds. Herein, our unit of analysis is the funding round, broken down into three main phases: seed, early stage and late stage.

Our main research aim is to examine the impact the COVID-19 crisis is having on entrepreneurial finance by volume, types of funding stages and types of firms. When looking at exogenous shocks, it is important to contextualise these events within their prevailing circumstances and trends so we also examine the wider trends affecting entrepreneurial finance more generally. The remainder of this article is structured as follows. We outline some exploratory empirical evidence examining the impact of the crisis on the market for entrepreneurial finance in the United Kingdom then offer brief conclusions and unpack future research issues to help guide further research.

## Empirical findings

**Figure 1.**
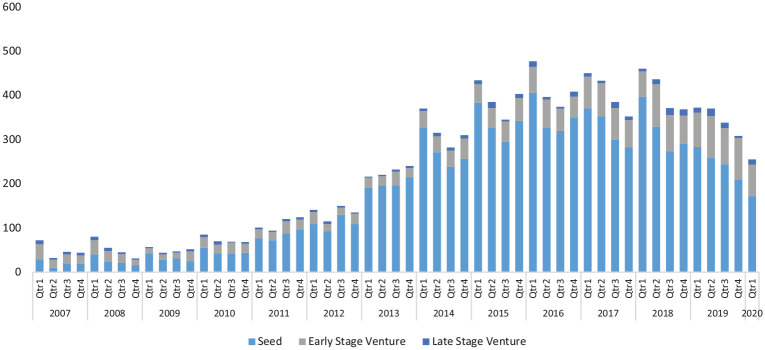
Number of UK deals by funding stage (2007–2020 Q1).

We now outline some indicative empirical findings from the research. What we can immediately observe is the considerable growth in this form of entrepreneurial finance since the time of the GFC (see Figure 1). Between 2007 and 2010, typically there has been a threefold increase in deal flow. The volume of new deals escalated particularly rapidly peaking in the first quarter of 2016 when there were almost 500 deals recorded. Since this time, there has been a significant decrease in the number of deals to around 350 per quarter which may owe to the impact of Brexit, which some argue has significantly reduced public sector co-investment in early-stage ventures (Brown et al., 2019). In terms of the size of these transactions, we have seen quite substantial growth in the value of these equity deals during this time period, especially in 2017 and 2019, when the value of deals increased by 62.5% and 25.6%, respectively. All in all, equity deals in the United Kingdom during the last decade are becoming significantly larger and lumpier.

So what has happened to the market for entrepreneurial finance since the outbreak of the COVID-19 pandemic? Overall, we can see in Table 1 that there has been a significant decrease in the levels of entrepreneurial finance deals in the first quarter of 2020 compared with the first quarter of 2019 to the first quarter of 2020 (31%). We have to go back to the first quarter of 2013 to witness such low levels of deals in quarter 1 2020 (see Table 1). These decreases occurred for seed and early-stage investments but not late-stage deals. Given the first quarter of the financial year is traditionally the strongest for equity deals,^4^ this would suggest further decreases throughout subsequent quarters in 2020 are highly likely.

**Table 1.** Number of deals by investment stage (2007–2020 Q1).

	Seed	Early stage	Late stage	Grand total
**2007**	78	91	25	194
**Qtr1**	29	34	9	72
**Qtr2**	10	18	4	32
**Qtr3**	20	20	6	46
**Qtr4**	19	19	6	44
**2008**	100	89	22	211
**Qtr1**	40	32	8	80
**Qtr2**	23	25	7	55
**Qtr3**	21	20	4	45
**Qtr4**	16	12	3	31
**2009**	126	59	15	200
**Qtr1**	43	11	3	57
**Qtr2**	28	12	4	44
**Qtr3**	31	13	3	47
**Qtr4**	24	23	5	52
**2010**	184	88	20	292
**Qtr1**	55	24	6	85
**Qtr2**	43	19	8	70
**Qtr3**	42	25	2	69
**Qtr4**	44	20	4	68
**2011**	333	89	17	439
**Qtr1**	76	21	4	101
**Qtr2**	72	19	3	94
**Qtr3**	88	27	5	120
**Qtr4**	97	22	5	124
**2012**	440	83	18	541
**Qtr1**	109	27	5	141
**Qtr2**	92	17	6	115
**Qtr3**	130	16	4	150
**Qtr4**	109	23	3	135
**2013**	797	96	15	908
**Qtr1**	191	23	2	216
**Qtr2**	196	21	3	220
**Qtr3**	196	31	5	232
**Qtr4**	214	21	5	240
**2014**	1092	156	29	1277
**Qtr1**	327	37	6	370
**Qtr2**	271	36	8	315
**Qtr3**	238	37	7	282
**Qtr4**	256	46	8	310
**2015**	1347	184	36	1567
**Qtr1**	383	42	9	434
**Qtr2**	327	44	14	385
**Qtr3**	295	46	4	345
**Qtr4**	342	52	9	403
**2016**	1401	220	34	1655
**Qtr1**	406	58	13	477
**Qtr2**	326	64	6	396
**Qtr3**	319	51	4	374
**Qtr4**	350	47	11	408
**2017**	1303	281	36	1620
**Qtr1**	370	72	8	450
**Qtr2**	352	75	6	433
**Qtr3**	299	72	14	385
**Qtr4**	282	62	8	352
**2018**	1288	300	47	1635
**Qtr1**	396	58	6	460
**Qtr2**	329	96	11	436
**Qtr3**	273	82	16	371
**Qtr4**	290	64	14	368
**2019**	995	348	45	1388
**Qtr1**	283	78	11	372
**Qtr2**	258	95	17	370
**Qtr3**	244	82	12	338
**Qtr4**	210	93	5	308
**2020**	172	71	12	255
**Qtr1**	172	71	12	255
**Grand total**	9656	2155	371	12,182

Qtr: quarter.

Given the real-time nature of the data, we can observe that the figure for the first two months of the second quarter in 2020 (i.e. April and May) has witnessed a significant drop compared to the previous years. This includes the period covering the lockdown enforced by the UK government which suspended trading in sizeable parts of the economy. In April and May 2020, there were only 134 new deals recorded compared to 286 in April–May 2018 and 245 in April–May 2019. In other words, the deal volume has roughly halved compared to previous years which may signify that the level of declines for the second quarter of 2020 could be much greater than in the first quarter. It should be noted however that the aggregate value of transactions in the first quarter of 2020 is higher compared with previous two years, which again suggests deal sizes are becoming much larger.

**Figure 2.**
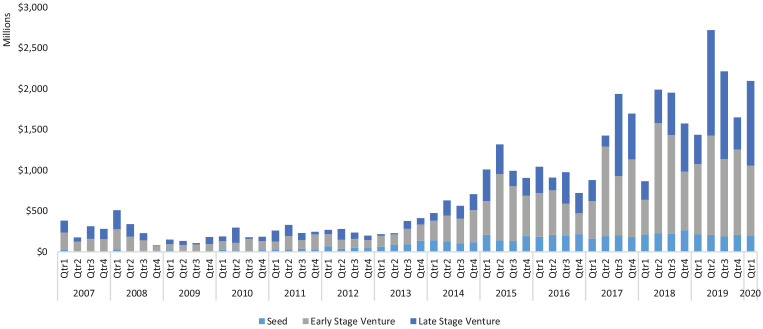
Volume of money raised by funding stage (2007–2020 Q1).

It is clear from the data that seed finance, which typically goes to the most early-stage entrepreneurial ventures, is by far the largest category of entrepreneurial finance by number of deals in the United Kingdom. In most years, as shown in Figures 1 and 2, this represents around three-quarters of all equity finance deals in the United Kingdom. We can see in Table 1 that seed finance has declined markedly since the first quarter of 2019 compared to the first quarter of 2020, a decrease of 39%, making it the deal type most heavily affected by the crisis. However, it is also abundantly clear that this form of early-stage finance is much lower in value than early-stage and late-stage deals. In most years, it comprises around 15% of all equity funding by value in the United Kingdom (see Figure 2).

While numerically dominant, seed finance is eclipsed by the value of transactions at early-stage and late-stage deals. We also see that during the last three years, late-stage deals have considerately increased in size. What is surprising is that late-stage deals actually increased between the first quarter of 2019 and that of 2020, suggesting that larger deals may be relatively insulated from the ensuing crisis. So, while seed finance is the category of finance most affected, this may potentially be offset by a slight growth of late-stage deals.

**Figure 3.**
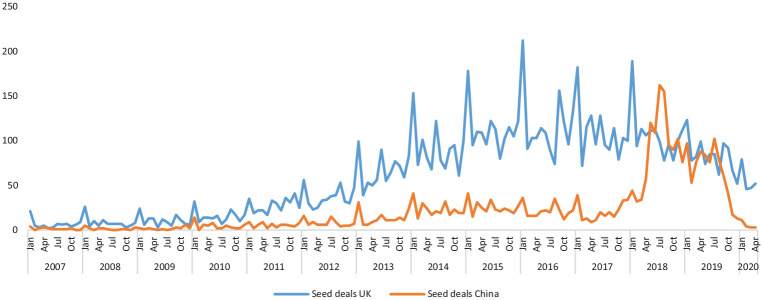
Number of seed deals from United Kingdom and China (January 2007–April 2020).

While space precludes a proper examination of how the crisis has precisely affected different countries, for comparative purposes, we also examined how China had been affected in the immediate aftermath of the COVID-19 pandemic. Given China was the first country to experience an outbreak of the pandemic, it could potentially offer interesting insights how other economies will also be negatively affected (Brown and Rocha, 2020). What this analysis reveals is a massive drop in volumes and value of equity finance during the first quarter of 2020 in China. Overall, the volume of entrepreneurial finance deals in China declined by 60% compared to the first quarter of 2019. This compares to a decrease of just over 30% reported above in the United Kingdom during the same period. In line with the United Kingdom, this decline in China was by far the steepest for seed stage deals. Figure 3 illustrates the differences in volumes of seed deals between the two economies since before the GFC. What this strongly suggests is that the market for seed finance has been hit the hardest in both countries, meaning nascent start-ups may be the most detrimentally affected firms during the current crisis period, irrespective of geographical location.

## Conclusion and future research

The COVID-19 pandemic has created a significant systemic economic shock, surpassing that of the GFC in 2007–2008 (Baker et al., 2020). Given its manifest importance to the economy, how the entrepreneurial finance market is affected by this chronic uncertainty will have a major and long-lasting effect on entrepreneurial and innovative activity for years to come (Howell et al., 2020). This article provides important timely insights into the uncertainty caused by the crisis by using a novel source of real-time data to investigate this topic. From our analysis, the United Kingdom seems to be significantly affected but the order of magnitude is considerably lower than in countries such as China (Brown and Rocha, 2020) and broadly in line with other major entrepreneurial finance markets such as the United States (Howell et al., 2020). This greater resilience probably owes to the more established nature and dense networks of equity finance actors within the United Kingdom’s entrepreneurial finance market compared to places like China. Technology may also be helping alleviate the reduced face-to-face interaction entailed by this form of finance. While the number of UK deals is considerably lower than in quarter 1 in 2019, the value of transactions has actually risen both, compared to the first quarter of 2019 and the final quarter of 2019. However, given the first quarter of the financial year is traditionally the strongest for equity deals, we are most likely to see a further continuation of this downwards trend for deal flow throughout the remainder of the year.

Overwhelmingly, the category of finance most adversely affected is seed finance deals for start-ups which decreased by almost 40% in the first quarter of 2020 compared to that of 2019 whereas late-stage deals have shown much greater resilience. It could be that later stage deals are associated with less risk as the investor already knows the firms and that the necessary face-to-face interaction has already occurred. What this means is that the entrepreneurial ventures most affected by the crisis are early-stage start-ups featuring the greatest levels of informational opacity. This shortage of finance for *de novo* ventures is of crucial importance because research shows that start-ups born during recessions not only start smaller, they tend to stay smaller in future years even when macroeconomic conditions improve (Sedláček and Sterk, 2017).

In terms of policy responses to support SME finances during the crisis, the overwhelming emphasis in the United Kingdom and other OECD economies has been support for debt finance in the form of loan guarantees and direct subsidised loans (OECD, 2020). Given the likely protracted dearth of new equity deals, the UK government may wish to incentivise equity investors during the crisis. Indeed, in recognition of the potential impact of the COVID-19 crisis for start-ups, the government has established a new Future Fund with a budget of £250 m which provides matched funding of between £250,000 and £5 m for equity funded ventures. While sizeable, there may need to be additional support measures to specifically target new seed stage deals, especially given the potential ramifications of ‘financial distancing’ between entrepreneurial firms and investors (Howell et al., 2020).

It also appears the Future Fund scheme may be incompatible with existing tax incentives such as the Enterprise Investment Scheme (EIS) and Seed Enterprise Investment Scheme (SEIS) designed to help small UK equity investors such as BAs invest in UK start-ups.^5^ This suggests this policy offering may be somewhat out of kilter with the current funding ecosystem in the United Kingdom. While rapid policy responses are needed to help mitigate crisis events (OECD, 2020), poorly designed policy instruments may accentuate (rather than reverse) the medium and longer-term effects of the current crisis.

Inevitably, exploratory empirical work such as this raises many more questions than it answers. Further investigation on the length of time taken for deals to be announced would provide clarity if later stage investments are more resilient in times of crisis or if this owes to lag effects masking results. It would be interesting to explore if VCs and BAs are returning to invest (returnee investors) in the same companies more often given face-to-face interactions may preclude new seed stage investments. Will larger VCs and BAs continue focusing on their existing portfolios and ignore future seed deals, further starving start-ups of cash over the longer-term? Conversely, will start-ups eventually overcome the lack of physical interaction or ‘mating’ opportunities with investors via online video pitches which are now commonplace in equity crowdfunding? How are different financial entrepreneurial ecosystems influenced by crisis events? Do some financial ecosystems have greater immunity to absorb shocks and major disturbances, as some suggest (Roundy et al., 2017), than others? While this commentary has focused on the supply of finance, some may wish to explore the bootstrapping or improvisational bricolage techniques entrepreneurs adopt during crisis periods to alleviate resource parsimony in innovative start-ups. We hope other scholars will seek answers to these crucial questions.

## Notes

1. Others have likened the levels of uncertainty to the Great Depression of 1929–1933 (Baker et al., 2020).

2.
https://about.crunchbase.com/blog/trends-european-vc-2019/

3. ‘The Crunchbase Data Difference’, Crunchbase, 3 April 2020, https://about.crunchbase.com/products/the-crunchbase-difference/

4. This has been the case in every year between 2007 and 2020 except for the years 2011, 2012 and 2013.

5.
https://seedlegals.com/resources/the-governments-future-fund-wont-help-uk-startups/

## References

[bibr1-0266242620937464] Baker SR Bloom N Davis SJ , et al. (2020) Covid-Induced Economic Uncertainty (No. w26983). Cambridge, MA: National Bureau of Economic Research.

[bibr2-0266242620937464] Bartik AW Bertrand M Cullen ZB , et al. (2020) How Are Small Businesses Adjusting to COVID-19? Early Evidence from a Survey (No. w26989). Cambridge, MA: National Bureau of Economic Research.

[bibr3-0266242620937464] Berger AN Udell GF (1998) The economics of small business finance: The roles of private equity and debt markets in the financial growth cycle. Journal of Banking & Finance 22(6–8): 613–673.

[bibr4-0266242620937464] Bernstein S Giroud X Townsend RR (2016) The impact of venture capital monitoring. The Journal of Finance 71(4): 1591–1622.

[bibr5-0266242620937464] Bertoni F Colombo MG Quas A (2015) The patterns of venture capital investment in Europe. Small Business Economics 45(3): 543–560.

[bibr6-0266242620937464] Block J Sandner P (2009) What is the effect of the financial crisis on venture capital financing? Empirical evidence from US Internet start-ups. Venture Capital 11(4): 295–309.

[bibr7-0266242620937464] Bonini S Capizzi V (2019) The role of venture capital in the emerging entrepreneurial finance ecosystem: Future threats and opportunities. Venture Capital 21(2–3): 137–175.

[bibr8-0266242620937464] British Business Bank (2020) Small business finance markets 2019/20. Available at: https://www.british-business-bank.co.uk/wp-content/uploads/2020/02/Small-Business-Finance-Markets-2019-20-report-FINAL.pdf

[bibr9-0266242620937464] Brown R Lee N (2019) Strapped for cash? Funding for UK high growth SMEs since the global financial crisis. Journal of Business Research 99: 37–45.

[bibr10-0266242620937464] Brown R Rocha A (2020) Entrepreneurial uncertainty during the Covid-19 crisis: Mapping the temporal dynamics of entrepreneurial finance. Journal of Business Venturing Insights 14: e00174.

[bibr11-0266242620937464] Brown R Liñares-Zegarra J Wilson JO (2019) The (potential) impact of Brexit on UK SMEs: Regional evidence and public policy implications. Regional Studies 53(5): 761–770.

[bibr12-0266242620937464] Brown R Mawson S Rowe A , et al. (2018) Working the crowd: Improvisational entrepreneurship and equity crowdfunding in nascent entrepreneurial ventures. International Small Business Journal 36(2): 169–193.

[bibr13-0266242620937464] Cassar G (2004) The financing of business start-ups. Journal of Business Venturing 19(2): 261–283.

[bibr14-0266242620937464] Clark C (2008) The impact of entrepreneurs’ oral ‘pitch’ presentation skills on business angels’ initial screening investment decisions. Venture Capital 10(3): 257–279.

[bibr15-0266242620937464] Conti A Dass N Di Lorenzo F , et al. (2019) Venture capital investment strategies under financing constraints: Evidence from the 2008 financial crisis. Research Policy 48(3): 799–812.

[bibr16-0266242620937464] Cowling M Liu W Ledger A (2012) Small business financing in the UK before and during the current financial crisis. International Small Business Journal 30(7): 778–800.

[bibr17-0266242620937464] Cowling M Rocha A Brown R (in press) Did you remember to save some of those internal funds for a rainy day? International Small Business Journal. 10.1177/0266242620945102 PMC8685737 35125601

[bibr18-0266242620937464] Cumming D Johan S Zhang Y (2018) Public policy towards entrepreneurial finance: Spillovers and the scale-up gap. Oxford Review of Economic Policy 34(4): 652–675.

[bibr19-0266242620937464] De Clercq D Sapienza HJ (2006) Effects of relational capital and commitment on venture capitalists’ perception of portfolio company performance. Journal of Business Venturing 21(3): 326–347.

[bibr20-0266242620937464] Demirgüç-Kunt A Peria MSM Tressel T (2020) The global financial crisis and the capital structure of firms: Was the impact more severe among SMEs and non-listed firms? Journal of Corporate Finance 60: 101514.

[bibr21-0266242620937464] Doern R Williams N Vorley T (2019) Special issue on entrepreneurship and crises: Business as usual? An introduction and review of the literature. Entrepreneurship & Regional Development 31(5–6): 400–412.

[bibr22-0266242620937464] Doshi H Kumar P Yerramilli V (2018) Uncertainty, capital investment, and risk management. Management Science 64(12): 5769–5786.

[bibr23-0266242620937464] Fraser S Bhaumik SK Wright M (2015) What do we know about entrepreneurial finance and its relationship with growth? International Small Business Journal 33(1): 70–88.

[bibr24-0266242620937464] Giupponi G Landais C (2020) Building effective short-time work schemes for the COVID-19 crisis. VOX, CEPR Policy Portal, 1 April. Available at: https://voxeu.org/article/building-effective-short-time-work-schemes-covid-19-crisis

[bibr25-0266242620937464] Gompers P Kovner A Lerner J , et al. (2008) Venture capital investment cycles: The impact of public markets. Journal of Financial Economics 87(1): 1–23.

[bibr26-0266242620937464] Goodell JW (2020) COVID-19 and finance: Agendas for future research. Finance Research Letters. Epub ahead of print 12 April 2020. DOI: 10.1016/j.frl.2020.101512 PMC7152896 32562472

[bibr27-0266242620937464] Hall BH Lerner J (2010) The financing of R&D and innovation. In: Hall BH Rosenberg N (eds) Handbook of the Economics of Innovation, vol. 1. Amsterdam: North Holland Publishing, pp.609–639.

[bibr28-0266242620937464] Herbane B (2010) Small business research: Time for a crisis-based view. International Small Business Journal 28(1): 43–64.

[bibr29-0266242620937464] Herbane B (2013) Exploring crisis management in UK small- and medium-sized enterprises. Journal of Contingencies and Crisis Management 21(2): 82–95. 10.1111/1468-5973.12371 PMC8441854 40477353

[bibr30-0266242620937464] Howell S Lerner J Nanda R , et al. (2020) Financial distancing: How venture capital follows the economy down and curtails innovation. Harvard Business School Working paper no. 20-115, 12 May. Available at: https://papers.ssrn.com/sol3/papers.cfm?abstract_id=3594239

[bibr31-0266242620937464] Huang L Knight AP (2017) Resources and relationships in entrepreneurship: An exchange theory of the development and effects of the entrepreneur-investor relationship. Academy of Management Review 42(1): 80–102.

[bibr32-0266242620937464] Kerr WR Nanda R Rhodes-Kropf M (2014) Entrepreneurship as experimentation. Journal of Economic Perspectives 28(3): 25–48.

[bibr33-0266242620937464] Kuckertz A Brändle L Gaudig A , et al. (2020) Startups in times of crisis – A rapid response to the COVID-19 pandemic. Journal of Business Venturing Insights 13: e00169.

[bibr34-0266242620937464] Lee N Sameen H Cowling M (2015) Access to finance for innovative SMEs since the financial crisis. Research Policy 44(2): 370–380.

[bibr35-0266242620937464] Martin R Berndt C Klagge B , et al. (2005) Spatial proximity effects and regional equity gaps in the venture capital market: Evidence from Germany and the United Kingdom. Environment and Planning A 37(7): 1207–1231.

[bibr36-0266242620937464] Nesta (2019) Paths to Scale: Finance Lessons from European Entrepreneurs. London: Nesta. Available at: https://media.nesta.org.uk/documents/Nesta_Scaling_Stories_Update_Web.pdf

[bibr37-0266242620937464] Organisation for Economic Co-operation and Development (OECD) (2020) Coronavirus (COVID-19): SME policy responses. Available at: http://www.oecd.org/coronavirus/policy-responses/coronavirus-covid-19-sme-policy-responses-04440101/

[bibr38-0266242620937464] Roundy PT Brockman BK Bradshaw M (2017) The resilience of entrepreneurial ecosystems. Journal of Business Venturing Insights 8: 99–104.

[bibr39-0266242620937464] Schwab A Zhang Z (2019) A new methodological frontier in entrepreneurship research: Big data studies. Entrepreneurship, Theory and Practice 43(5): 843–854.

[bibr40-0266242620937464] Sedláček P Sterk V (2017) The growth potential of startups over the business cycle. American Economic Review 107(10): 3182–3210.

[bibr41-0266242620937464] Shane S Cable D (2002) Network ties, reputation, and the financing of new ventures. Management Science 48(3): 364–381.

[bibr42-0266242620937464] Wenzel M Stanske S Lieberman M (2020) Strategic responses to crisis. Strategic Management Journal. Epub ahead of print 1 April 2020. DOI: 10.1002/smj.3161.

[bibr43-0266242620937464] Wishart M (2018) Business Resilience in a SME Context: A literature Review. Enterprise Research Centre. Available at: https://www.enterpriseresearch.ac.uk/wp-content/uploads/2018/07/Resilience-review-Final.pdf

[bibr44-0266242620937464] Wright M Hart M Fu K (2015) A nation of angels: Assessing the impact of angel investing across the UK. Report by the Enterprise Research Centre. Available at: https://www.enterpriseresearch.ac.uk/wp-content/uploads/2019/11/ERC-Angels-Report.pdf

